# Supplementary material for: Engineered dsRNA–protein nanoparticles for effective systemic gene silencing in plants
Source: Hortic Res. 2024 Feb 22;11(4):uhae045. doi: 10.1093/hr/uhae045 (PMC11497610; doi:10.1093/hr/uhae045)
Supplement: Web_Material_uhae045 [file web_material_uhae045.docx]

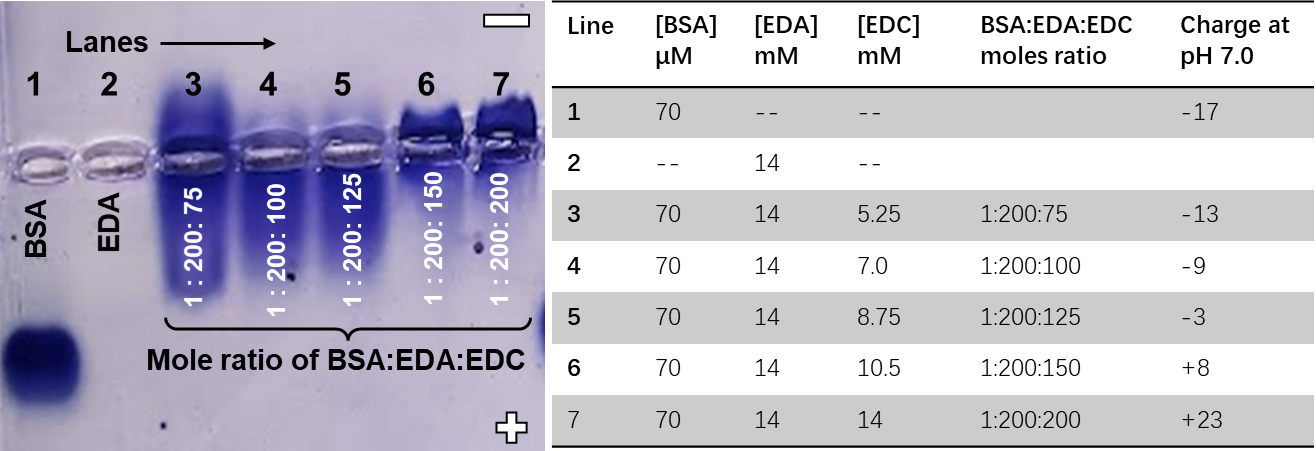


**Fig. S1** Agarose gel electrophoresis of BSA and BSA-EDA conjugates stained with Coomassie blue. The samples were spotted at the center of the gel and migrated to up or down depending on their charge. Lane 1: BSA, Lane 2: EDA, Lane 3-7: reaction mixture of BSA, EDA and EDC in the mole ratios as indicated in each lane. Electrophoresis was done at pH 7.0 in 40 mM Tris-acetate buffer.


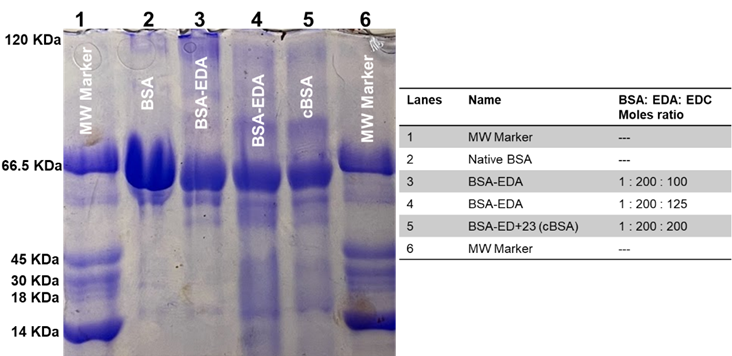


**Fig. S2** The SDS-PAGE of the BSA-EDA conjugates. Lanes 1 and 6: molecular weight markers; Lane 2: unmodified BSA; Lane 3-6: BSA-EDA conjugates at increasing stochiometries.


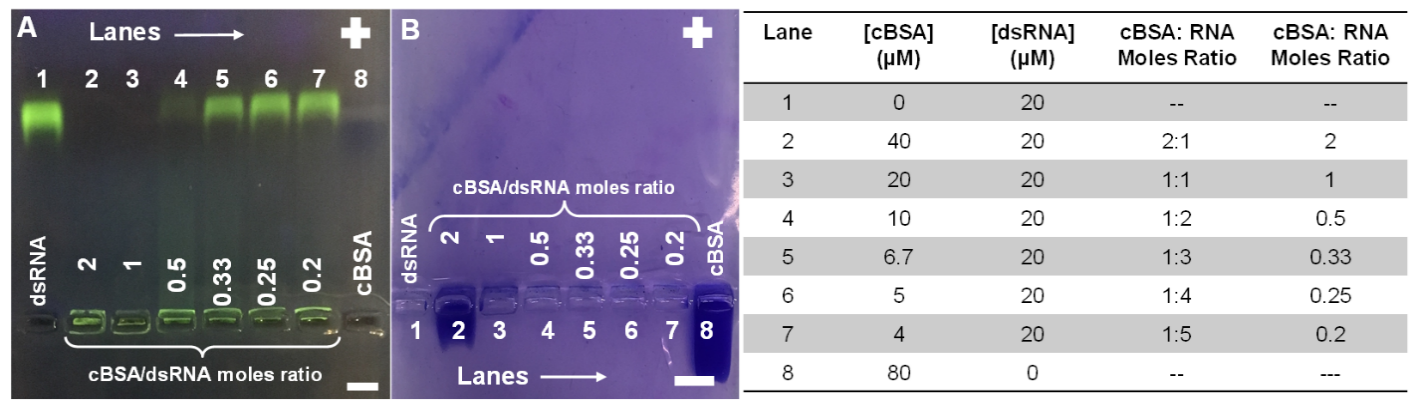


**Fig. S3** cBSA titrations with dsGUS RNA as monitored in agarose gel electrophoresis. (**A**) Stained with SYBR green dye to see RNA bands, and (**B**) Stained with Coomassie blue to see protein bands. The numbers above the wells in both gels represent the mole ratios of cBSA to dsGUS RNA, Lane 1: dsGUS RNA; Lanes 2-7: increasing mole ratio of cBSA to dsGUS RNA; Lane 8: cBSA, no RNA. The gel was run at pH 7.0 in 40 mM Tris-acetate buffer.


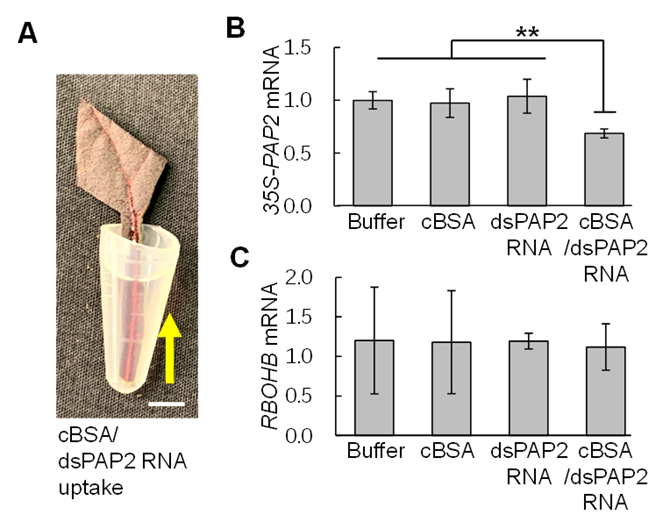


**Fig. S4** Systemic silencing effects of cBSA/dsPAP2 RNA nanocomplexes on expression of constitutively active *35S-PAP2* gene in tobacco. (**A**) cBSA/dsPAP2 RNA nanocomplexes were fed into leaf tissues from basal ends of leaf petioles. The feeding lasted for 72 hours and each cm^2^ of leaf tissues received about 0.4 mL solution of cBSA/dsGUS RNA nanocomplexes (1 x 10^-6^ M). Scale bar, 0.5 cm. (**B**-**C**) qPCR analysis of expression of the *35S-PAP2* gene in the treated leaves shows the *PAP2* transcript (**B**) was reduced by cBSA/dsPAP2 RNA nanocomplexes, while expression of an oxidative stress inducible *RBOHB* gene (**C**) was not affected. *Elongation Factor 1α* gene (*NtEF1α*), a housekeeping gene, was used for normalizing the expression levels of all genes. ** indicates significant differences at p<0.01 by ANOVA. The data presented are means±AVEDEV, which have been calculated from a minimum of six biological replicates.

**Table S1** The sequence information of double-strand GUS RNA. Two pieces of dsGUS RNA (dsGUS1 and dsGUS4), fully complementary to the *DR5-GUS* and *35S-GUS* mRNA in the transgenic tobacco and poplar, were synthesized by Genolution (Seoul, Korea).

| **dsRNA** | **Length** | **Sequence** |
| --- | --- | --- |
| dsGUS1 RNA | 126 bp | CGACGCUCACACCGAUACCAUCAGCGAUCUCUUUGAUGUGCUGUGCCUGAACCGUUAUUACGGAUGGUAUGUCCAAAGCGGCGAUUUGGAAACGGCAGAGAAGGUACUGGAAAAAGAACUUCUGGC |
| dsGUS4 RNA | 127 bp | UGAUUAACCACAAACCGUUCUACUUUACUGGCUUUGGUCGUCAUGAAGAUGCGGACUUACGUGGCAAAGGAUUCGAUAACGUGCUGAUGGUGCACGACCACGCAUUAAUGGACUGGAUUGGGGCCAA |

**Table S2** The sequence information of primers for qPCR analysis.

| **Primer** | **Sequences** |
| --- | --- |
| GUS-F | GATATCTACCCGCTTCGCGTCG |
| GUS-R | CATGTTCATCTGCCCAGTCGAG |
| NtANN12-F | CTTCTCTGCCCTTGTAACTAT |
| NtANN12-R | CAACCGCTACAAGGGTGATTA |
| RBOHB-F | TTTCTCTGAGGTTTGCCAGCCACCACCTAA |
| RBOHB-R | GCCTTCATGTTGTTGACAATGTCTTTAACA |
| NtEF1α-F | TGAGATGCACCACGAAGCTC |
| NtEF1α-R | CCAACATTGTCACCAGGAAGTG |
| PtEF1β-F | GACAAGAAGGCAGCGGAGGAGAG |
| PtEF1β-R | CAATGAGGGAATCCACTGACACAAG |
